# Inter-study reproducibility of cardiac MRI in free breathing patients at rest for the evaluation of regional myocardial perfusion

**DOI:** 10.1186/1532-429X-18-S1-W32

**Published:** 2016-01-27

**Authors:** Travis DeSa, Jeremy D Collins, James C Carr, Michael Markl, Kai Lin

**Affiliations:** Radiology, Northwestern University Feinberg School of Medicine, Chicago, IL USA

## Background

In the early stages of some cardiovascular diseases (CVDs), myocardial ischemia only occurs in a limited area. Thus, accurate detection of regional myocardial perfusion abnormalities is important for diagnosis. Recently, myocardial perfusion using MRI has become a promising non-invasive method to evaluate myocardial ischemia in CVDs. However, the reproducibility of MRI for assessment of regional perfusion has not been studied. This knowledge gap could affect the use of Cardiac MR in clinical practice. To address this clinical need, we evaluate the reproducibility of MRI perfusion in 12 healthy volunteers.

## Methods

12 healthy volunteers (10 males, mean age 52 ± 17 years old, Body weight: 93 ± 20 Kg) were recruited after IRB approval. Written informed consent was obtained. MRI scans were performed at 1.5 T (Aera, Siemens). Each participant underwent two cardiac MRI perfusion scans (steady-state free precession) with the same protocols separated by a 2-week interval. Perfusion data were acquired at base, mid, and apex levels of the left ventricle (LV) after injection of a single dose gadopentetate dimeglumine (Magnevist, 0.1 ml/kg). Motion Correction Imaging was used to account for cardiac and respiratory motion. The parameters were as follows: FOV = 360 × 360 mm2, Slice thickness = 8 mm, TR/TE/flip angle = 168/1.14 ms/12degree. Measurements = 60

The images were transferred to a computer (Siemens Leonardo Syngo) for analysis using ARGUS software. A single reviewer drew the borders (endo and epi) of the LV at each slice for each study. The peak signal intensity, slope of the signal change, and time to peak perfusion (TTP) were evaluated in individual myocardial segments (based on AHA-16-segment model).

The reproducibility of peak signal intensity, slope, and TTP was assessed using intraclass correlation coefficient (ICC) and coefficient of variation **(**COV). Bland-Altman plots were also applied to show inter-study variations.

## Results

**Figure 1:**
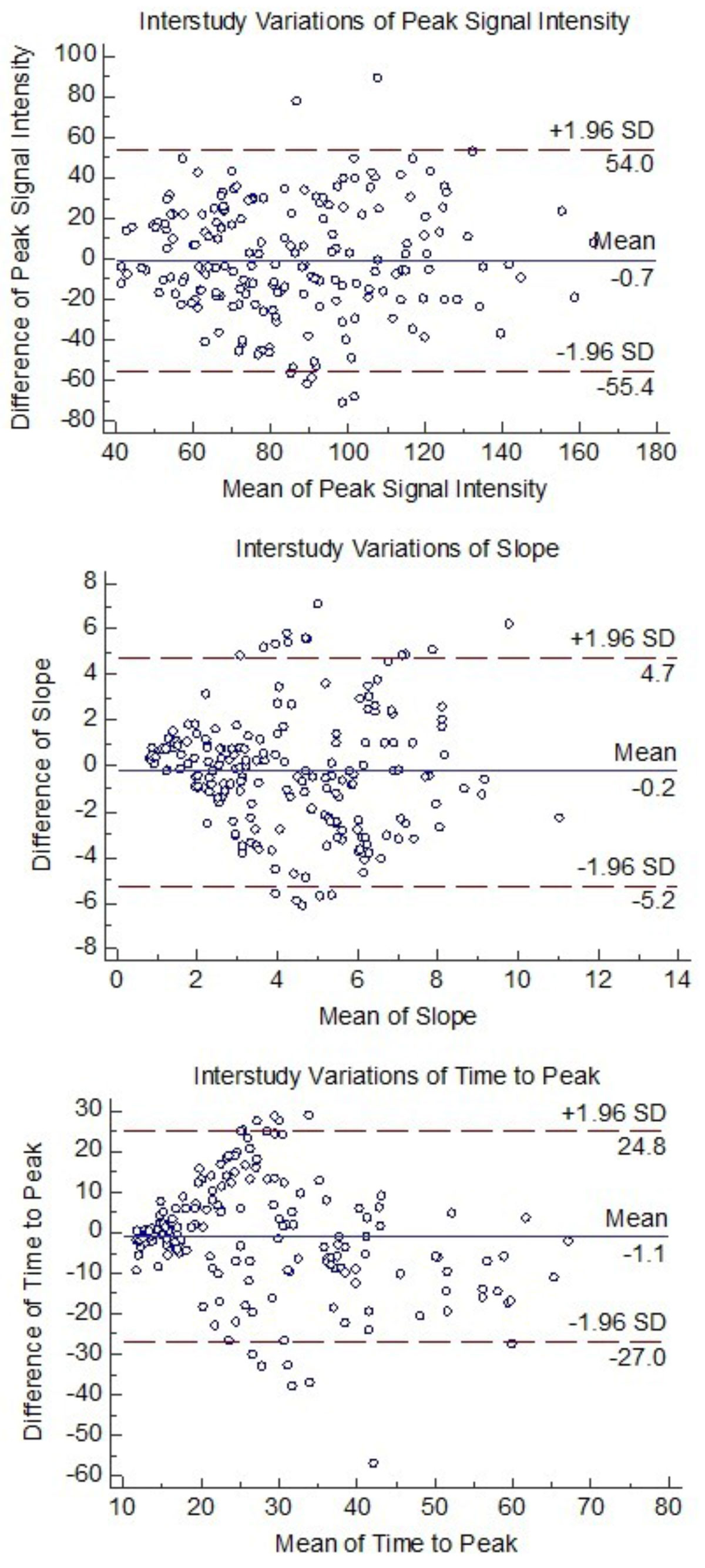
**Bland-Altman plots showing inter-study variations**.

Cardiac MR was successful in 12 volunteers twice and resulted in 192 pairs of data points for each of the three parameters. As summarized in Table 1, the first scan perfusion mean peak intensity was 85.27 ± 29.06 and that of the second scan was 85.98 ± 28.75. The first scan slope mean was 4.18 ± 2.46 and that of the second scan was 4.42 ± 2.54. The first scan TTP mean was 26.23 ± 13.01 and that of the second scan was 27.34 ± 16.18. Between scans and re-scans, on a per segment basis, peak signal intensity, slope, and TTP demonstrated moderate to good agreement with ICC values of 0.554, 0.52, and 0.735 respectively (all, p < 0.001). In addition, the peak signal intensity, slope, and TTP were moderately variable with COV values of 23.01%, 41.96%, and 34.93% respectively. Bland-Altman plots are shown in Figure 1.

**Table 1:** Table 1

	Scan 1	Scan 2	ICC	COV (%)
Peak Intensity	85.27 ± 29.06	85.98 ± 28.75	0.554	23.01
Slope	4.18 ± 2.46	4.42 ± 2.54	0.52	41.96
TTP	26.23 ± 13.01	27.34 ± 16.18	0.735	34.93

## Conclusions

Cardiac MR is a reproducible technique for quantitative assessment of segmental myocardial perfusion at rest during quiet breathing. However, technical improvements in image acquisition and processing are needed to further lower its inter-study variability.

